# The Necessity of a Colonoscopy after an Acute Diverticulitis Event in Adults Less Than 50 Years Old

**DOI:** 10.7759/cureus.5666

**Published:** 2019-09-16

**Authors:** Daniel C Alcantar, Claudia Rodriguez, Richard Fernandez, Sanjay Kumar, Christine Junia

**Affiliations:** 1 Internal Medicine, Macneal Hospital, Berwyn, USA

**Keywords:** diverticulitis, colonoscopy, colorectal cancer

## Abstract

Introduction

Acute diverticulitis is defined as a macroscopic inflammation of a diverticulum or diverticula. Approximately, 4.0% of patients with diverticulosis present with an acute diverticulitis event: with the incidence increasing in the younger patient population. According to the American Gastrointestinal Association, a colonoscopy should be performed six to eight weeks after resolution of acute diverticulitis. The purpose of this study is to determine if there is malignancy after an acute diverticulitis event in adults less than 50 years old.

Methods

A retrospective chart review study was performed at Loyola MacNeal Hospital in Berwyn, Illinois. Patients between the ages of 18 and49 years with acute diverticulitis were identified. Of the identified patients, those who underwent colonoscopy after a computed tomography (CT)-verified acute diverticulitis event were recorded. Colonoscopy findings, as well as pathology results, were recorded.

Results

A total of 295 patient presented with diverticulitis. Of these 295 patients, 111 patients underwent colonoscopy post diverticulitis event. Of the 111 patients, 86 were after uncomplicated event and 25 were after a complicated diverticulitis event. Pre-malignant tubular adenomas were found in 12.8% (11/86) of patients with acute uncomplicated diverticulitis and 24.0% (6/25) of the patients with complicated diverticulitis. No cases of neoplasm were found.

Conclusion

Of the 111 patients who underwent colonoscopy after an acute diverticulitis event, no malignancy was found in patients less than 50 years of age.

## Introduction

Acute diverticulitis is defined as a macroscopic inflammation of a diverticulum or diverticula. Approximately, 4.0% of patients with diverticulosis present with a diverticulitis event [1]. Since 2012, acute diverticulitis has become the third most-common gastrointestinal diagnosis, with an increase of 41.0% from 2000 and an estimated cost of 2.6 billion dollars per year [2]. Interestingly, the rates of admission have been increasing, especially in the younger population [3].

According to the American Gastrointestinal Association (AGA) guidelines, it is recommended to perform a colonoscopy six to eight weeks after resolution of acute diverticulitis [1]. Per the AGA, the reason behind this recommendation was based on an observational study which showed a small number of colorectal cancers and advanced adenomas [1]. However, the association of colorectal cancer and diverticulitis still remains uncertain. Multiple studies have investigated the need for colonoscopy after an acute diverticulitis event, and of these studies, the results have varied. 

To our knowledge, only one study has investigated the younger patient population; unfortunately, this study was based in Asia, where right-sided diverticulosis is more common [4]. The purpose of this study is to determine the risk of malignancy after an acute diverticulitis event in adults less than 50 years old. By reviewing these data, we can then further investigate current guidelines post diverticulitis event and see if they are applicable to the entire population, particularly to our younger patient population. 

## Materials and methods

A retrospective chart review study was performed at Loyola MacNeal Hospital, Berwyn, Illinois. All patients admitted with an acute diverticulitis event from January 2007 to December 2017 were included. Patient records were identified using the ICD-9 and ICD-10 codes for acute diverticulitis. 

Patients between the ages of 18-49 years old with acute diverticulitis were identified using computed tomography (CT). CT findings that confirmed acute diverticulitis included: colonic wall thickening and stranding of pericolonic fat [5]. Of the identified patients, they were subcategorized into uncomplicated diverticulitis, which is defined as localized inflammation, and complicated diverticulitis, which is defined as inflammation associated with an abscess, phlegmon, fistula, obstruction, bleeding, or perforation [6]. Those patients who underwent colonoscopy were identified and the biopsy results were recorded. Patients without CT verification of diverticulitis, and patients greater than 50 years old were excluded.

This work was presented at Digestive Disease Week (DDW) in San Diego, California in May 2019. The abstract was then published in the American Gastroenterology Association journal of abstracts (Gastroenterology, 2019) [7].

## Results

**Figure 1:**
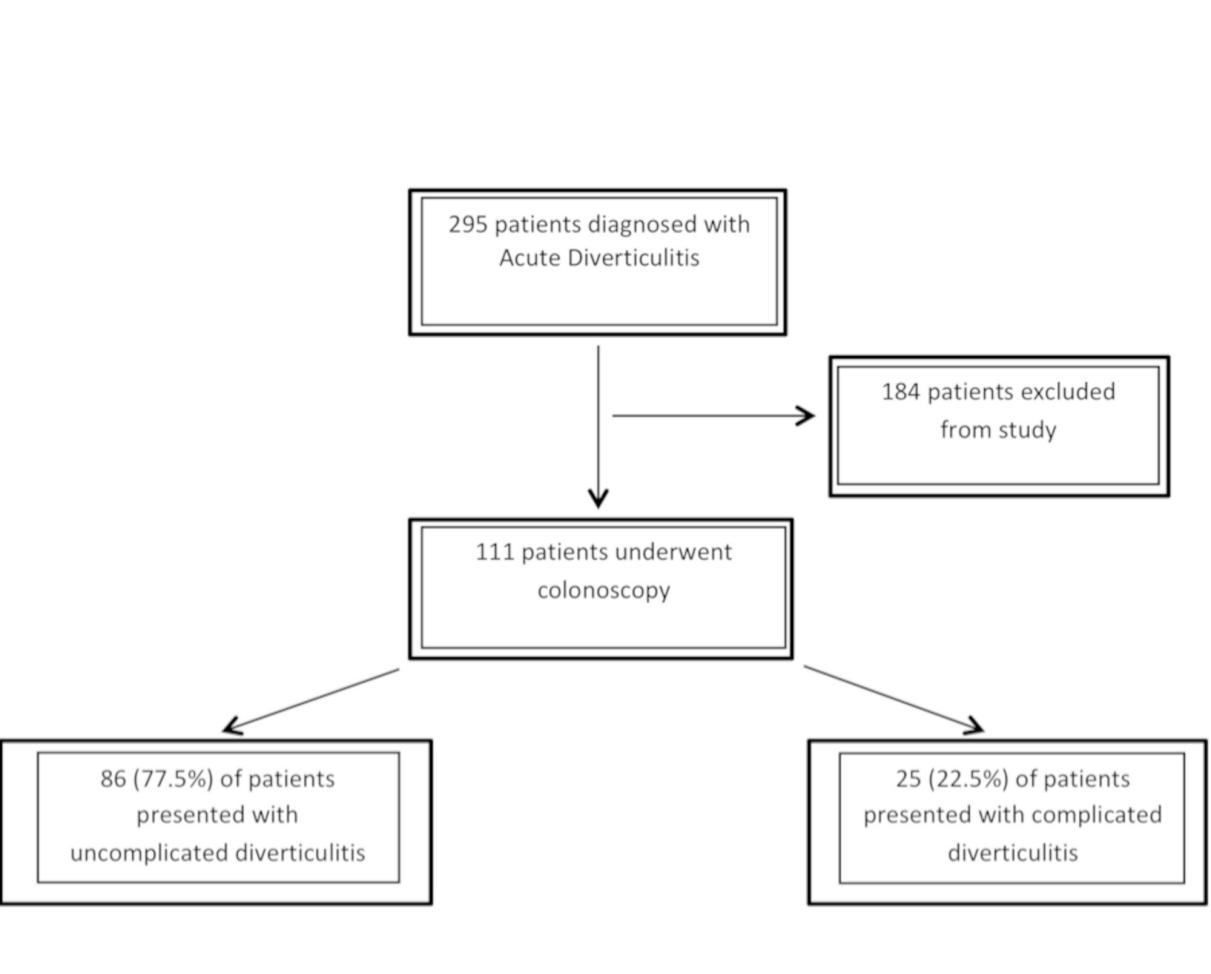
Flow of patients through the study

A total of 295 patients were identified between the dates of 2007 and 2017. Of these 295 patients, 111 (37.6%) of these patients underwent colonoscopy post diverticulitis event, excluding 184 patients. The mean age of the patient population was 40.70, with 67 (60.3%) of patients being male and 44 (39.6%) of patients being female. Among the 111 patients who presented with acute diverticulitis, 86 (77.5%) patients presented as an uncomplicated event and 25 (22.5%) presented as complicated (figure 1).

Of the 86 patients with uncomplicated diverticulitis, 11 patients were found to have tubular adenomas, 18 patients presented with a hyperplastic polyp, and 48 of the patients did not have biopsy performed. Of the 25 patients with complicated diverticulitis, six patients presented with tubular adenomas, four patients presented with a hyperplastic polyp, and eight patients had no biopsy performed. Of these patients, zero cases of neoplasm were found (Table 1).

**Table 1:** Pathologic results from biopsy samples (n =111)

Finding	Uncomplicated, No. (%)	Complicated, No. (%)
Adenocarcinoma	0 (0)	0 (0)
Adenoma	11 (12.8)	6 (24.0)
Benign	18 (20.9)	4 (16.0)
No biopsy	45 (55.1)	8 (32.0)

## Discussion

Currently, there have been several studies that have investigated the risk of malignancy associated with acute diverticulitis. Agarwal AK et al. performed a systemic review for which it reviewed four large studies and found that 1.2-2.1% of patients had malignancy and 19.5-20.2% of patients were found to have nonmalignant polyps [8]. Sharma et al. performed a similar but larger systemic review for which 11 studies where investigated and discovered a crude risk of 1.2% chance of malignancy post acute diverticulitis event; however, a mean of 19.5% of patients presented with nonmalignant polyps [9]. When comparing our data with both large systemic reviews, our results were different. No malignancy was identified in our study and 15.3% (including complicated and uncomplicated) of our patient population had nonmalignant polyps. These differences could be secondary to the patient population investigated as well as a large amount of patients excluded (n=184) due to loss of care. When comparing our study to both systemic reviews, we collected data on patients less than 50 years of age, which could lead a smaller amount of patient population; whereas the systemic reviews included all adults greater than 18 [8-9]. In these systemic reviews, the age of colorectal malignancy was not recorded, making it difficult to identify and age predilection for malignancy among the included patients, but it was noted that the median age of acute diverticulitis was 57-64 years old. Lastly, we characterized each polyp identified and subcategorized them into an uncomplicated or complicated event. This was not performed in both systemic reviews.

To date, there continues to be conflicting data in regards to the increased risk of malignancy in patients with complicated diverticulitis versus uncomplicated. Sharma et al. systemically reviewed six studies with a pooled population of 1497 with uncomplicated diverticulitis [9]. Out of the 1497 patients, colorectal malignancy was found in five patients with a crude proportional rate of a colorectal malignancy in 0.7% and 15.1% (138) patients were found to have nonmalignant colorectal polyps. Patients with complicated diverticulitis were also investigated. After reviewing five studies, a pooled population of 79 patients was found to have complicated diverticulitis. Six malignancies were identified with a proportional pooled rate of 10.8%; whereas, subgroup analysis for nonmalignant polyps was not performed secondary to low patient data. When comparing our data to the systemic review, again it was noted that no malignancy was identified in either the complicated diverticulitis or uncomplicated diverticulitis group. Furthermore, our study identified adenomatous polyps as well as benign polyps. 

Based on our findings and results from other studies, the conclusions have been vague. Which leads us to the question, is colonoscopy necessary post diverticulitis event particularly among a younger patient population? Colonoscopy is an invasive procedure, though low-risk; still has complications. Rutter et al. performed a retrospective cohort study to look for adverse events after screening and follow-up colonoscopy [10]. In this large study of 43,456 patients, it was found that the 4.7 serious adverse events per 1000 screening colonoscopies and 6.8 per 1000 follow-up colonoscopies can occur. Because of the risks of colonoscopy, multiple studies have looked at imaging as a possible alternative. Chintapalli et al. performed a retrospective study, where it was found that pericolonic lymph nodes and luminal mass findings on CT were significant for colon cancer [11]. However, after further investigating these results prospectively, the CT findings were unequivocal. Öistämö et al. retrospectively investigated both CT and MRI as possible tools to diagnose colon cancer [12]. It was found that the MRI had a sensitivity and specificity of 100% of diagnosing colon cancer; however, this study was fairly small, with a total of only 16 patients investigated. Lastly, CT-colonography (CTC) showed promising results in regards to identifying diverticular disease with a sensitivity and specificity of 99.0% and 67.0%; unfortunately, it had a poor sensitivity (47.0%) and specificity (75.0%) for detection of polyps [13]. Although these studies are encouraging, the results are equivocal and lack power [10-13]. Because of this, endoscopy with biopsy should continue to be standard of care after an acute diverticulitis event. 

To our knowledge, there has been only one study performed by Dedrick et al. [4]. This retrospective study investigated whether or not colonoscopy is necessary in patients less than 50 years. Unfortunately, this study was performed in Asia, where right-sided colonic diverticulitis is more commonly found. In this study, only 27 patients underwent colonoscopy post diverticulitis event, among those patients, no evidence of malignancy was identified; however, 11.0% of their patient population had neoplastic potential. When looking into western countries where left-sided diverticulitis is common, Kim et al. and Pisanu et al. performed retrospective chart reviews for which again no evidence of malignancy was found among patients less than 50 years old [14-15]. It was noted that 15.0% of the patient population <50 were found to have adenomas. In our study, we found similar results where no malignancy was detected on follow-up colonoscopy and noted that 15.3% (n =17), including both uncomplicated and complicated, were found to have tubular adenomas, which is comparable to the other studies [9, 14-15].

The limitations of this study include the total patient population. Gathering data from a smaller community hospital, a wider time frame was made in order to identify a higher number of cases. However, despite such a wide time frame, the total number of patients identified were minor. Furthermore, of the patients identified, more than 50.0% of patients were lost to follow up, which could lead to miss diagnoses of malignancy. Furthermore, the demographic characteristics, location of diverticulitis, and other confounding factors such as a family history of malignancy were not studied.

## Conclusions

There have been multiple studies that have debated whether or not patients should undergo colonoscopy after a diverticulitis event. However, of these studies; adults of all ages were included. There have been limited retrospective studies on the younger patient population which our project also aimed to further evaluate. In summary, we identified no cases of malignancy in patients with acute diverticulitis who were less than 50 years of age. Given the similarities of our findings with other studies, but the inherent limitations of these retrospective reviews, we would recommend further research among the younger patient population to help adjust guidelines based on their potentially different malignancy risk profile.

## References

[1] Strate LL, Peery AF, Neumann I (2015). American gastroenterological association institute technical review on the management of acute diverticulitis. Gastroenterology.

[2] Bharucha AE, Parthasarathy G, Ditah I (2015). Temporal trends in the incidence and natural history of diverticulitis: a population-based study. Am J Gastroenterol.

[3] Amersi F, Agustin M, Ko CY (2005). Colorectal cancer: epidemiology, risk factors, and health services. Clin Colon Rectal Surg.

[4] Chan DKH, Tan KK (2017). There is no role for colonoscopy after diverticulitis among Asian patients less than 50 years of age. Gastrointest Tumors.

[5] Kim MJ, Woo YS, Kim ER (2014). Is colonoscopy necessary after computed tomography diagnosis of acute diverticulitis. Intest Res.

[6] (2019). American Family Physician. https://www.aafp.org/afp/2013/0501/p612.pdf.

[7] Alcantar D, Kumar S, Fernandes Almeida R, Rodriguez CG, Junia CC, Sprang ME (2019). Is colonoscopy necessary after an acute diverticulitis event in adults less than 50 years old. Gastroenterology.

[8] Agarwal AK, Karanjawala BE, Maykel JA, Johnson EK, Steele SR (2014). Routine colonic endoscopic evaluation following resolution of acute diverticulitis: is it necessary. World J Gastroenterol.

[9] Sharma P, Eglinton T, Hider P, Frizelle F (2013). Systematic review and meta-analysis of the role of routine colonic evaluation after radiologically confirmed acute diverticulitis. Ann Surg.

[10] Rutter C, Johnson E, Miglioretti DL, Mandelson MT, Inadomi J, Buist DS (2012). Adverse events after screening and follow-up colonoscopy. Cancer Causes Control.

[11] Chintapalli KN, Chopra S, Ghiatas AA, Esola CC, Fields SF, Dodd GD (1999). Diverticulitis versus colon cancer: differentiation with helical CT findings. Radiology.

[12] Öistämö E, Hjern F, Blomqvist L, Von Heijne A, Abraham-Nordling M (2013). Cancer and diverticulitis of the sigmoid colon. Differentiation with computed tomography versus magnetic resonance imaging: preliminary experiences. Acta Radiol.

[13] Chabok A, Smedh K, Nilsson S, Stenson M, Påhlman L (2013). CT colonography in the follow-up of acute diverticulitis: patient acceptance and diagnostic accuracy. Scand J Gastroenterol.

[14] Pisanu A, Vacca V, Reccia I, Podda M, Uccheddu A (2013). Acute diverticulitis in the young: the same disease in a different patient. Gastroenterol Res Pract.

[15] Krones CJ, Klinge U, Butz N (2006). The rare epidemiologic coincidence of diverticular disease and advanced colonic neoplasia. Int J Colorectal Dis.

